# Swimming and Persons with Mild Persistant Asthma

**DOI:** 10.1100/tsw.2007.221

**Published:** 2007-08-17

**Authors:** Mirjana Aranđelović, Ivana Stanković, Maja Nikolić

**Affiliations:** Faculty of Medicine, University of Niš, Serbia

**Keywords:** asthma, swimming, lung function

## Abstract

The aim of our study was to analyze the effect of recreational swimming on lung function and bronchial hyperresponsiveness (BHR) in patients with mild persistent asthma. This study included 65 patients with mild persistent asthma, who were divided into two groups: experimental group A (n = 45) and control group B (n = 20). Patients from both groups were treated with low doses of inhaled corticosteroids (ICS) and short-acting β2 agonists salbutamol as needed. Our program for patients in group A was combined asthma education with swimming (twice a week on a 1-h basis for the following 6 months). At the end of the study, in Group A, we found a statistically significant increase of lung function parameters FEV1 (forced expiratory volume in 1 sec) (3.55 vs. 3.65) (p < 0.01), FVC (forced vital capacity) (4.27 vs. 4.37) (p < 0.05), PEF (peak expiratory flow) (7.08 vs. 7.46) (p < 0.01), and statistically significant decrease of BHR (PD_20_ 0.58 vs. 2.01) (p < 0.001). In Group B, there was a statistically significant improvement of FEV1 3.29 vs. 3.33 (p < 0.05) and although FVC, FEV1/FVC, and PEF were improved, it was not significant. When Groups A and B were compared at the end of the study, there was a statistically significant difference of FVC (4.01 vs. 4.37), FEV1 (3.33 vs. 3.55), PEF (6.79 vs.7.46), and variability (p <0.001), and statistically significantly decreased BHR in Group A (2.01 vs. 1.75) (p < 0.001). Engagement of patients with mild persistent asthma in recreational swimming in nonchlorinated pools, combined with regular medical treatment and education, leads to better improvement of their parameters of lung function and also to more significant decrease of their airway hyperresponsiveness compared to patients treated with traditional medicine

## INTRODUCTION

Asthma is an inflammatory airway disease associated with bronchial hyperresponsiveness (BHR) and reversible airflow obstruction. The Global Initiative for Asthma (GINA) recommends that patients with persistent asthma should use an inhaled corticosteroid (ICS) and short-acting β2 agonist bronchodilator as needed. ICS therapy can improve parameters of lung function and reduce BHR in patients with persistent asthma[1]. The quality of treatment and quality of life of people with asthma are focused on enabling them to experience all aspects of life, with sport and swimming as an important factor. Guidelines for asthma therapy recommend swimming, but people with asthma believe that you should not exercise.

Several Olympic swimmers (such as Mark Spitz, Dawn Fraser, and Jon Hendricks) were asthmatic, but that was not a barrier to their physical fitness[2,3]. Swimming is connected to proper physical and psychological development, and evidence shows that regular swimming combined with asthma education can increase the lung volume and help to develop good breathing techniques. Swimming is excellent for improving muscle tone and general fitness, and is also great fun for all people with asthma[4,5,6].

William Osler, the Father of American Medicine, stated, "The practice of medicine is an art, based on science."The conclusion of Kraft was that no single treatment may be absolutely correct to treat mild, persistent asthma[7,8].

The aim of our study was to analyze the effect of recreational swimming on the parameters of lung function and BHR in patients with mild persistent asthma.

## METHODS

This study was performed as a prospective, randomized, parallel group trial at the departments of the Health Center, University of Nis. One year ago, we studied 70 patients with mild, persistent asthma selected according to GINA with documented treatment with low doses of ICS and short-acting β2 agonists. The patients of the experimental group began to practice swimming twice a week on a 1-h basis for the following 6 months, and treatment with low doses of ICS and short-acting β2 agonists salbutamol (Group A, n = 45). The program for patients in Group A with asthma had combined swimming with asthma education. This group used an indoor swimming pool with natural warm water without chlorination, located in a recreational spa center in the vicinity of their home. The patients in Group B (n = 20) were treated with low doses of ICS and short-acting β2 agonists salbutamol. Five did not stay until the end of the study. Therefore, 45 patients in Group A and 20 in Group B completed the study.

At the beginning and the end of the study, the spirometry and bronchoprovocative test with histamine were measured in all the patients. The skin prick test was performed in order to examine the atopic status. Also, the patients in Group A had asthma education before the testing begun (within asthma school).

### Spirometry

The parameters of lung function: forced expiratory volume in 1 sec (FEV1), forced vital capacity (FVC), and FEV1/FVC × 100 were recorded using a flow-volume spirometer (Jaeger) according to the recommendations of the European Respiratory Society[9].

### Bronchoprovocative Test

Aerosol of histamine was delivered by a dosimeter device (APS). The challenge started with a histamine dose of 15 µg, which was then increased in doubling doses until FEV1, measured within 1 min of histamine inhalation, decreased 20% or more when compared with the baseline of FEV1. The dose of histamine causing a 20% decrease in FEV1 ($PD_{20}$ histamine) was calculated by interpolation of the dose response curves[10,11].

### Skin Tests

The atopic status was assessed by the skin prick test to a panel of five common aeroallergens and defined as positive of ≥3 mm to one or more allergens.

## Asthma Education

Asthma education was conducted within an asthma school, which comprised information on therapy, breathing, diet, mental hygiene, smoking, physical activities, and quality of life through related courses.

## RESULTS

Table 1 shows the baseline demographic characteristics of all the participants. The groups were comparable, and there was no significant difference in terms of age, sex, atopic status, and smoking characteristics. All 45 subjects in group A (34 female, 11 male; mean age 33.07 ± 9.81 years) and 20 subjects in group B (15 female, five male; mean age 33.55 ± 10.88) were treated with ICS, beclomethasone dipropionate 250 µg/day and short-acting β2 agonists (Ventolin) as needed. In addition, the patients of the experimental group began to practice swimming twice a week on a 1-h basis for the following 6-month trial (see Table 1).

**TABLE 1**
**Demographic Characteristics of the Patient**

| | Group A (n = 45) | Group B (n = 20) |
|---|---|---|
| Age | 33.07 ± 9.81 | 33.55 ± 10.88 |
| Sex (M/F) | 34/11 | 15/5 |
| Atomic | 69% | 63% |
| Smoking | | |
| Never | 78.8% | 81.0% |
| Ex-smoker | 21.2% | 19.0% |
| Current | 0% | 0% |

At the beginning of the study, FEV1, FVC, and FEV1/FVC were similar in both groups. After 6 months, group A showed statistically significant improvement of FEV1 (3.55 vs. 3.65) ($p < 0.01$), FVC (4.27 vs. 4.37; $p < 0.05$), PEF (7.08 vs. 7.46; $p < 0.01$). Group B showed statistically significant improvement of FEV1 (3.29 vs. 3.33; $p < 0.05$); although FVC, FEV1/FVC, and PEF were improved, the change was not significant. When group A and B were compared at the end of the study, there was a statistically significant difference for FVC, FEV1, and PEF variability ($p < 0.001$). The changes in the parameters of lung function are shown in Table 2.

At the end of the study, there was significant improvement in reduced BHR in group A ($PD_{20}$ 0.58 vs. 2.01; $p < 0.001$) and in group B ($PD_{20}$ 061 vs. 1.75) (see Table 3). After comparing BHR between group A and B at the end of study, we found a statistically significant difference ($p < 0.001$), with BHR significantly lower in group A. The analysis of BHR in relation to atopic status showed that at the beginning of the examination in both groups, there was no significant change in relation to the atopic status. At the end of the examination, in group A, both in the atopic ($PD_{20}$ 0.45 vs. 1.66; $p < 0.01$) and the nonatopic patients ($PD_{20}$ 1.01 vs.2.28; $p < 0.001$) showed a significant decrease of BHR. In group B, there was a statistically significant decrease of BHR in the atopic (0.61 vs.1.44; $p < 0.05$) and in nonatopic patients (1.05 vs. 1.92; $p < 0.01$) (see Table 4).

**TABLE 2**
**Lung Function, Initially and at the End of the Study**

| | Group A | | Group B | |
|---|---|---|---|---|
| | **Baseline** | **End of Study** | **Baseline** | **End of Study** |
| FVC (L) | 4.27 ± 0.97 | 4.37 ± 0.98*** | 3.99 ± 0.92 | 4.01 ± 0.93[a] |
| FEV1 (L/s) | 3.55 ± 0.85 | 3.65 ± 0.86** | 3.29 ± 0.78 | 3.33 ± 0.84***,[a] |
| FEV1/FVC | 83.8 ± 5.63 | 84.4 ± 4.83 | 82.8 ± 4.88 | 83.0 ± 5.94 |
| PEF (L/S) | 7.08 ± 1.87 | 7.46 ± 1.67** | 6.72 ± 1.17 | 6.79 ± 1.45[a] |

*$p < 0.001$, **$p < 0.01$, ***$p < 0.05$, [a]$p < 0.001$(group A vs. B at the end of study).

**TABLE 3**
**$PD_{20}$, Initially and at the End of the Study**

| | Group A | | Group B | |
|---|---|---|---|---|
| | **Initially** | **End of Study** | **Initially** | **End of Study** |
| $PD_{20}$ | 0.58 ± 0.70 | 2.01 ± 1.68* | 0.61 ± 0.48 | 1.75 ± 0.22**,[a] |

*$p < 0.001$, **$p < 0.05$. [a]$p < 0.001$ (group A vs. B at the end of the study).

$PD_{20}$ = provocative dose of histamine causing a 20% fall in FEV1.

**TABLE 4**
**$PD_{20}$, Initially and at the End of the Study when Compared to the Atopic Status**

| | **Initially** | **End of Study** |
|---|---|---|
| Group A | | |
| Atopic | 0.45 ± 0.42 | 1.66 ± 1.48** |
| Nonatopic | 1.01 ± 1.21 | 2.28 ± 1.86* |
| Group B | | |
| Atopic | 0.61 ± 0.48 | 1.44 ± 0.22*** |
| Nonatopic | 1.05 ± 0.96 | 1.92 ± 0.58** |

*$p < 0.001$, **$p < 0.01$, ***$p < 0.05$.

## DISCUSSION

The golden standard of asthma treatment for patients with persistent asthma should be ICS as the first-line maintenance treatment and short-acting β2 agonists as needed. In our 6-month prospective examination, the patients in the experimental group with the addition of swimming exercise showed improved clinical status. The analysis of lung function parameters at the end of the study showed increase of all the analyzed spirometric parameters in both examined groups. Better lung function after 6-month ICS treatment has been found in other studies[1,12], but our research was focused on the group that completed an asthma instruction program as well as recreational swimming. This group showed statistically important increase in FVC, FEV1, and PEF compared to controls. Similar results were found by others[13,14], and studies with children and adolescents with asthma have shown that practicing of swimming can lead to significant improvement in FEV1 and PEF[5,6,7,15,16]. Wardell and Isbister[17], in their study on swimming programs for children with asthma (38 boys and 35 girls with an average of 2–4 years in the program), suggested justification for the program. For many people, the program can improve quality of life and asthma management, and reduce medication, visits to physicians, and hospitalizations[17]..

There are many different opinions regarding the positive influence of swimming in patients with asthma; for example, that nitrogen trichloride, which is present in indoor chlorinated pools, has a very negative influence on breathing organs, and also the claim that there is recorded professional asthma in employees in charge of the cleaning, supervision, and maintenance of swimming pools[18,19,20]. Patients in our study used a recreational indoor swimming pool with natural warm water without chlorination. The good news is that swimming is one of the best forms of exercise for children with asthma as it is less likely to trigger asthma symptoms than other forms of exercise[21,22].

The objective of our study was to analyze BHR initially and at the end of the study. In all the patients, a nonspecific bronchoprovocative test was performed with increasing histamine doses and the provocative dose, which led to a 20% decrease of FEV1 ($PD_{20}$). The results showed a significant impact of swimming on BHR. Group A showed a significant decrease of BHR with the implication that ICS and recreational swimming were beneficial on BHR in patients with persistent asthma. There is evidence that physical activity may prevent or modify airway inflammation[23,24]. Further studies are needed to determine the mechanisms underlying this association.

The atopic status and BHR are genetically determined, but it is also well known that BHR is present with both atopic and nonatopic asthma. In their study, Sandfort and Pare[25] showed that BHR on metacholine is under genetic control of about 66%[25]. The objective of our study was to analyze BHR initially and at the end of the study in relation to the atopic status. The results showed that at the beginning of the study $PD_{20}$, there was no statistically significant difference between atopic and nonatopic patients, which marks the presence of BHR in nonatopic asthma and points to different mechanisms of its occurrence. Our results were in accordance with the results of Mochizuki et al.[26], who found no difference in degree of BHR between atopic and nonatopic asthma patients. These results could be explained by Kraft's theory that no single treatment may be absolutely correct to treat mild, persistent asthma that, therefore, recreational swimming can be recommended. At the end of the study in the atopic and the nonatopic patients in group A, there was a significant decrease of BHR; in group B, there was also an decrease of BHR, but it was significantly decreased in the nonatopic group B.

Atopic asthma is now the most common chronic childhood disease in the U.S. and many other industrialized countries. The cause for the rise is unclear, though many hypotheses have been put forth, but some have speculated that the increased use of indoor chlorinated swimming pools by children could be a reason. Chlorination products interact with atopic status to increase the risk of childhood asthma (adjuvant effect). Some have suggested that chlorine by-products, such as trichloramine, disrupt the protective epithelial barriers of the respiratory tract, allowing allergens to enter the lungs[27,28,29,30]. Our data suggested that low ICS dose, swimming practice, and asthma education over a period of 6 months led to a significant decrease of BHR, which is an indirect measure of inflammation. For recreational swimming, the patients used an indoor swimming pool with natural warm water without chlorination and this insight could be beneficial for the society in general, but especially for architects who plan the building of swimming pools in recreational centers. The number of people with asthma is growing each day, especially within the pediatric population, which indicates the need for building special recreational pools for those individuals inclined to have allergic response and those with allergic diseases of airways. The best solution is a combination of the following factors: existence of swimming pools with natural spa water with regulated flow combined with excellent education regarding the proper way of swimming pool usage, high conscience of people who use these swimming pools, and an understanding by society that this kind of need exists for one part of the population.

The results of the study were limited by the short period of the examination and the relatively small number of observed patients, but we hope that our already-started detailed examinations with longer trial period will result in more precise evidence.

## CONCLUSION

Engagement of patients with mild, persistent asthma in recreational swimming in nonchlorinated pools, combined with regular medical treatment and education, leads to better improvement of their parameters of lung function and also to a more significant decrease of their airway hyperresponsiveness compared to patients treated with traditional medicine.
